# A systematic review on the relationship between antisocial, borderline and narcissistic personality disorder diagnostic traits and risk of violence to others in a clinical and forensic sample

**DOI:** 10.1186/s40479-016-0046-0

**Published:** 2016-10-13

**Authors:** Joe Lowenstein, Charlotte Purvis, Katie Rose

**Affiliations:** Pan Dorset Pathfinder Service, Dorset Healthcare NHS Foundation Trust, St. Ann’s Hospital, 69 Haven Road, Canford Cliffs, Poole, Dorset BH13 7LN UK

**Keywords:** Personality disorder, Violence, Forensic, Risk assessment, Systematic review

## Abstract

Risk assessments identify the presence of a Personality Disorder diagnosis as relevant to future violence. At present, risk assessments focus on the presence of the disorder rather than identifying key traits related to risk. Systematic searches of three databases were conducted from January 2000 until August 2014. Of 92,143, 15 studies met the inclusion criteria. A lack of empirical research was found focusing on individual traits; instead most considered PD diagnosis as a sole entity. A preliminary model has been developed detailing the link between potential interactions of diagnostic traits and risk of violence. Recommendations for future research are made.

## Background

### Personality disorders and risk

The process of assessing and managing risk continues to evolve, with the hope of ever increasing accuracy. This is never truer than in the domain of Personality Disorder (PD), with current approaches to risk assessment “failing to provide a systematic framework for assessors to use to make sense of the heterogeneous presentations typically found in individuals with Personality Disorder and violence” ([33], pp.610).

Davison and Janca [8] emphasise the need to employ an integrated risk framework that considers the diagnostic traits of PDs and their co-morbidity with other known risk factors. Although the HCR-20 V3 [12] includes the concept of PD in its assessment proforma, there is the need for a more expansive approach, as it fails to attend to individual traits which are considered to be linked to violence and are thus relevant when developing a formulation for the management in the long and short term. It also regards Antisocial Personality Disorder (ASPD) and/or psychopathy as the leading PD diagnosis to consider in risk management.

Identifying relevant personality traits that are empirically linked to violence, would be a more comprehensive method of formulating individualised risk assessment and management plans, than purely relying on a diagnostic entity which can often be heterogeneous. Focusing on PD diagnoses alone in risk assessment is precarious as it fails to take into account the complexity of a clinical diagnosis, and risks the oversight of relevant information [10] such as severity of personality difficulties, protective personality traits and treatment responsiveness.

### Defining violence

Violence has been explicitly identified as a significant public health problem and currently, a number of widely used definitions and concepts of violence are used within the public health field [39]. For the purposes of this paper, violence is defined as “actual, attempted, or threatened infliction of bodily harm of another person” ([12], pp.2), and includes serious psychological harm, defined as causing “fear of physical injury, as well as other emotional, mental, or cognitive consequences that subsequently interfere with health or well-being” ([12], pp.3). This definition is taken from the widely used HCR-20 Version 3 violence risk assessment tool and is consistent with the World Health Organisation’s definition of ‘interpersonal violence’ [30]. Five key aspects of this definition are deemed important by Douglas and colleagues:

1. The focus is on behaviour that is, or can be linked to physical or serious psychological harm as previously defined. Acts serious enough for the perpetrator to be charged are considered violent.
2. The physical or serious psychological harm must impact on others aside from the perpetrator (e.g. self-harm may be included if others also suffer harm).
3. Diverse behaviour including complete, incomplete and planned/attempted acts may be included as well as a single incident versus a more chronic pattern.
4. The behaviours above are purposeful and engaged in with the general aim of inciting physical or serious psychological harm on others.
5. The behaviour is not sanctioned either through legal means or through consent from a victim (e.g. Mixed martial arts).

Overall, Douglas et al. [12] acknowledge the difficulties in defining the term (and the implications that this has on risk assessment) but suggest an overall summary of violence as occurring when; a person engaged in an act (or omission) with some degree of wilfulness that caused or had the potential to cause physical or serious psychological harm to another person or persons.

### Disorders and violence

When considering the occurrence of violence, it is imperative to recognise that violence results from a complex interplay of factors, not one entity, such as a PD diagnosis. It is known that violent individuals have a more complex psychopathology than non-violent individuals [50], and it is essential to consider the interplay between environmental and organic factors [14].

Evidence indicates that individuals with a PD diagnosis are at a significantly greater risk of aggressive and violent behaviour [46]. However, it is important to recognise that the above research states at ‘greater risk’, and is not deterministic that all individuals with a diagnosis of PD behave violently nor present serious danger to society. In fact, Coid et al. [4, 5]) identified that only 11 % of those with a PD diagnosis reported violent behaviour over the past 5 years in comparison to 7 % of the population without a diagnosis of PD.

It is commonplace for certain PD clusters or diagnostic traits to be associated with an increased propensity to engage in violent behaviour, however “uncertainties regarding the nature and extent of this relationship persist” [21]. This is particularly due to the inherent difficulties of overlapping symptoms in PDs, the reliance on cross-sectional assessment methods, which fail to capture the stability of PD and violence over time, and the use of small or unrepresentative samples. In addition, there is a need to take into account the discrepancy in personality symptoms from self-reports, interviews and assessments, and even the assessment instrument itself [43].

Nestor [36] reviewed the relationship between violence among persons with certain psychological disorders and identified four fundamental personality dimensions that may increase the risk of violence: 1) impulse control, 2) affect regulation, 3) threatened egotism or narcissism, and 4) paranoid cognitive personality style. It was concluded that two of these dimensions—impulse control and affect regulation—are likely compromised by all psychological disorders linked to violence. These dimensions may play a more specific role in violence and may be particularly important as additional critical features in explaining acts of violence in individuals with cluster B PDs.

### Cluster B personality pathology and violence

Coid et al. [4, 5] identified that men and women, with traits of Cluster B PD, rather than Cluster A or C, were 10 times more likely to have received criminal convictions and almost 8 times more likely to have served time in prison. Despite this, it is important to remain mindful of the tendency for over diagnosis within forensic and prison populations [26]. Studies have demonstrated a link between Cluster B pathology and increased anger, aggression and violence; Posternak and Zimmerman [38]) identified that patients with Cluster B personality pathology were 4.6 times more likely to report anger and had a stronger association with angry aggression.

Empirical data has focused predominantly on Borderline Personality Disorder (BPD) and ASPD, whilst data on the remaining PDs from Clusters A to C, is sparse [16]. This appears to be due to the assumption that BPD and ASPD diagnoses are more prominently linked to an elevation in criminal risk [35]. Hiscoke et al. [24] found that individuals with a diagnosis of ASPD had 3.7 times higher reconviction rates for attempted or completed murder, manslaughter, assault, robbery, or rape. Violence in the context of a diagnosis of ASPD has been identified primarily as instrumental, whereas violence associated with BPD diagnosis appears to be emotionally driven [11], which supports the theory that BPD and aggression may be mediated by difficulties with emotion regulation [40].

Baumeister et al. [3]) identified that incarcerated violent offenders demonstrated significantly elevated levels of pathological narcissism, in particular on the subscales of entitlement and superiority, which relates to the construct of “threatened egotism”. Warren et al. [48] found a powerful relationship between Narcissistic Personality Disorder (NPD) diagnosis and violent behaviour in females; specifically they were eight times more likely to have a current conviction for a violent offence (including homicide). Pathological narcissism and low levels empathy for the victims, both features of psychopathy, significantly increase the risk of serious violence [16]. However, there remains limited empirical evidence that considers the contribution of NPD diagnostic traits as a relevant risk factor for violent behaviour [32].

In order to advance in the domain of risk assessment, it is necessary to establish a more comprehensive understanding of the specific features of personality that may influence risk. This may then prevent loss of critical information [19] that may result in the inaccurate labelling of individuals as ‘risky’ solely based on a PD diagnosis. Tyrer et al. [43] posit that it is a mistake to link the concept of “dangerousness” in risk management to the severity of a PD diagnosis, as it can be present in other mental disorders and in non-pathological individuals.

## Method

### Search strategy

A systematic literature search was conducted using OvidSP, Science Direct and Pubmed to scrutinise the relationship between cluster B PD diagnostic traits and violence. Only articles from 2000 to present were included. Using the Boolean reference, the term “Violence” AND each of the 25 traits of ASPD, BPD and NPD were searched in the above databases. The search terms were replicated from Warren and South [49], which is discussed in the review. If the above search terms hit > 500 results, the term “Personality Disorder” was added to the search phase. If this failed to reduce the results to < 500, then “Cluster B Personality” was added to the search.

### Study selection

#### Exclusion criteria

Studies with only Cluster A PDs, Cluster C PDs and Histrionic Personality Disorder were excluded. Studies not written in English, published prior to the year 2000, and that did not use a clinical or criminal sample were also excluded.

### Data collection & analysis

**Fig. 1:**
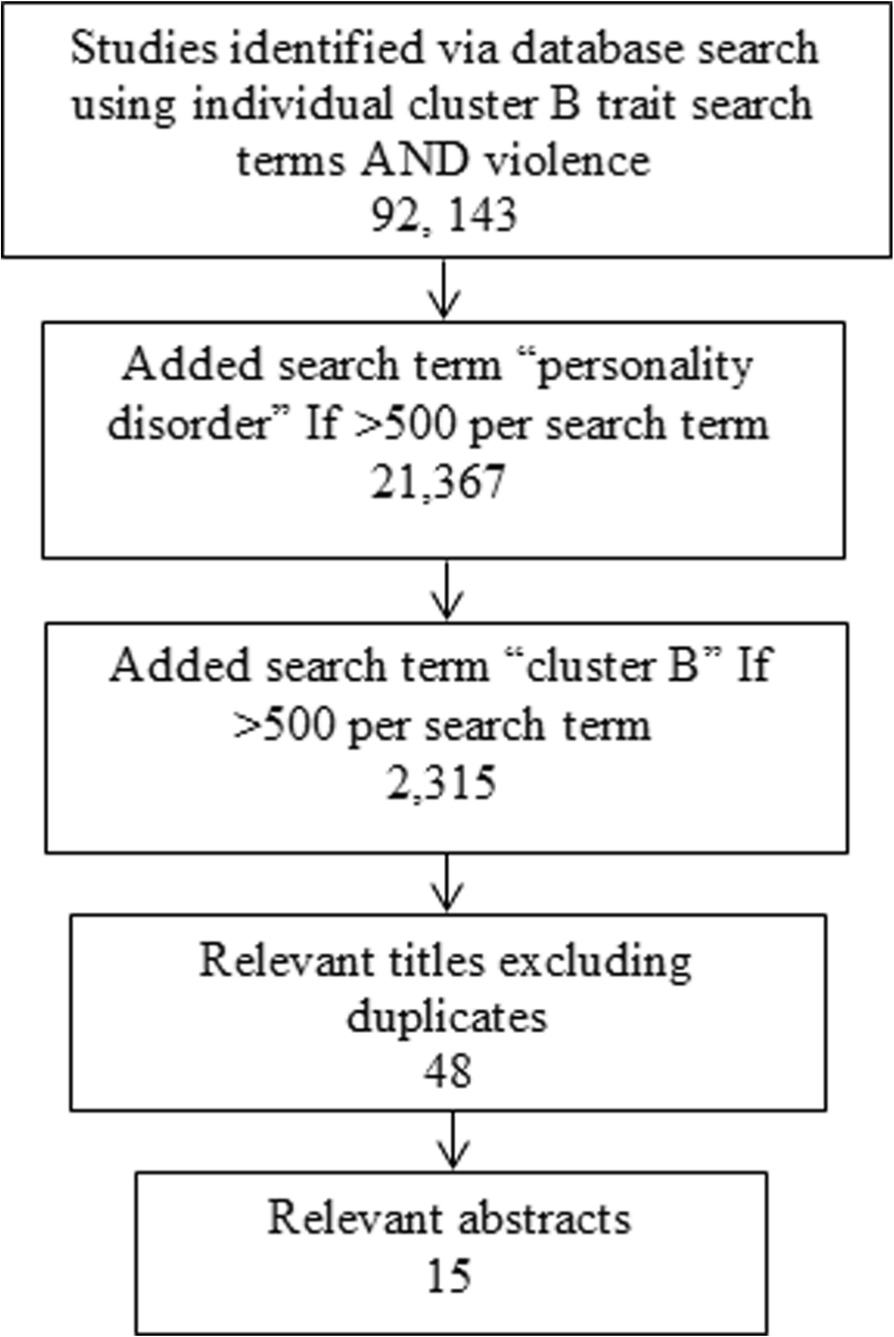
Paper identification process

Pertinent studies were identified from abstracts. Full papers were assessed according to the selection criteria and specificity and sensitivity. Randomised controlled trials and Pilot studies were included in the review. See Fig. 1 flow chart paper identification.

## Results

Table 1 summarises the content of the papers that met the inclusion criteria for systematic review.

**Table 1:** Summary of papers reviewed

	Authors	Sample size (*N*)	Trait(s) identified	Violence definition
1	Ullrich et al. [44]).	1136	grandiose	Serious violence defined as: (1) Batteries resulting in physical injury the use of a weapon. (2) Sexual assaults. (3) Threats made with a weapon.
2	Fisher and Hall [18].	120	sense of entitlement	Type of violence not specified.
3	Warren and South [49].	250	all	Violence defined as: capital murder, homicide, second degree murder, accomplice to murder, attempted homicide, manslaughter, abduction, assault, malicious wounding, felony assault, hurling missile, simple assault, abuse and cruelty, child abuse.
4	Lawson and Brossart [31].	132	avoid abandonment	Mild physical aggression (e.g., control physically, “push, grab) and severe physical aggression (e.g., choked, strangled, or beat up).
5	Goldenson et al. [22]).	65	unstable relationships	Type of violence not specified.
6	Seidel et al. [41]).	60	lack of empathy	Type of violence not specified.
7	Shorey et al. [42]).	80	anger	Domestic violence defined as: offenses ranging from assault and battery, stalking, harassment, and violation of orders of protection.
8	Scott et al. [40].	150	affective instability	Psychological Aggression defined as: Yelling, verbal insults) and Physical Assault defined as: shoving, slapping.
9	Mauricio et al. [34].	192	anger	Psychological Violence defined as: Emotional/verbal abuse (withholding emotional support, verbal attacks, and humiliation). Physical abuse not defined.
10	Fossati et al. [19]).	461	aggressiveness	Type of violence not specified but aggression divided into: Physical, verbal, anger; and hostility.
11	Day et al. [9]).	45	lack of empathy	Type of violence not specified.
12	Cunha and Gonçalves [7].	187	paranoid ideation	Type of violence not specified.
13	James and Seager [27].	40	impulsivity	Type of violence not specified.
14	Warren et al. [48]).	261	recklessness	Violence defined as: capital murder, homicide, second degree murder, accomplice to murder, attempted homicide, manslaughter, abduction, assault, malicious wounding, felony assault, hurling missile, simple assault, abuse and cruelty, child abuse.

### Ullrich et al. [44]

The Ullrich et al. [44] study was initially screened into the review due to its reference to ‘grandiose delusions’ in the abstract, which suggested a link to the NPD personality trait of “grandiose”. However on further exploration, the ‘grandiose delusions’ referred to a psychotic delusion of having special gifts or powers. Despite this, the study remains pertinent as it argues that emotion can contribute to the formation and maintenance of delusions [20], which may account for the fine line between grandiose thoughts and grandiose delusions. Ullrich et al. [44]) reanalysed data from the MacArthur Violence Risk Assessment Study using a prospective approach to investigate associations between specific delusions and violence in 1136 male and female civil psychiatric inpatients after discharge. Results confirmed previous research findings that experiences of delusions 10 weeks prior to discharge did not predict violence in the subsequent 10 weeks. The study did however, identify a pathway between the experience of grandiose delusions, such as belief in possessing special gifts/powers, and serious violence: This same delusion, when associated with elation or anger, demonstrated a direct pathway to serious violence, irrespective of affect due to the belief.

### Fisher & Hall [18]

In the absence of an appropriate psychometric for measuring the concept of ‘entitlement’, Fisher and Hall [18] constructed and validated a ‘Sense of Entitlement Questionnaire’ (SOEQ), informed by the results of a thorough literature review which identified 10 appropriate domains. The SOEQ was administered to 120 Australian male incarcerated offenders: 60 non-violent offenders and 60 violent offenders. Results indicated that violent offenders demonstrated an inflated sense of entitlement in both attitude and behaviour that, when violated, were more likely to result in violent behaviour. The main limitation of the study, which the authors identified, was that ‘length of stay’ in prison was not measured. This may have biased the results, as offenders who had served the longest time indicated that their sense of entitlement had significantly reduced since sentencing; Zamble [51] coined this phenomenon the “maturity factor”. Fisher and Hall [18], highlighted the necessity for future research to investigate whether an ‘inflated sense of entitlement’ has capacity for change, and could thus be considered a criminogenic need.

### Warren & South [49]

Warren and South [49] used a sample of 261 female offenders at a high security prison to investigate how PD symptoms related and predicted patterns of criminal and violent behaviour. Results showed a significant relationship between the scales and the antisocial, borderline and narcissistic factors. In fact the presence of antisocial, borderline and narcissistic personality traits all showed a significant relationship with perpetrating threats and physical assaults in the prison environment. Interestingly, women with narcissistic personality traits, co-morbid with less distinct antisocial personality traits, were consistently related to behavioural indices of threatening and violent behaviour. These individuals were identified as having elevated levels of aggression and anger, exaggerated but fragile self-esteem and a pervasive lack of remorse. Overall, they found that women with a diagnosis of NPD, or NPD comorbid with other PD disgnoses were eight times more likely to engage in violence, particularly murder. This current study highlights the opportunity for the study to be replicated with male prisoners to investigate whether similar results may occur.

### Lawson & Brossart [31]

Lawson and Brossart [31] researched the strength of (predominantly) antisocial and borderline diagnostic personality traits and interpersonal difficulties, as mediators between attachment and intimate partner violence severity in 132 men on probation for intimate partner violence. Results showed that through the mediation of interpersonal difficulties, avoidant attachment predicted the severity of physical partner violence, which supports previous research [13]. The authors concluded that interpersonal difficulties in the form of hostile-dominance may be a more effective and applicable psychopathology construct, than personality traits, for use as a risk predictor for intimate partner violence.

### Goldenson et al. [22]

Goldenson et al. [22] examined personality traits, attachment style and symptoms of trauma in 33 female offenders receiving court-ordered treatment and 32 non-offending females receiving psychological treatment. Goldenson et al. [22] found that female offenders had a higher array of Cluster B Personality psychopathology, reported a greater number of traumatic experiences and exhibited less secure attachment styles. 48 % and 39 % of the female offender group met the clinical cut-off for Antisocial and Borderline MCMI-III subscales and 48 % met the clinical cut-off for the Narcissism subscale. Interestingly, mean score group differences were found to be significant for the Antisocial and Borderline subscales, but not for the Narcissistic subscale. Limitations identified were; the small sample size, the fact the groups were unmatched in terms of compulsory treatment and reliance on self-reports rather than structured clinical interviews. The authors recommended replication of the study using a within-group method, to assess for variations in personality pathology within a female court-ordered offender group.

### Seidel et al. [41]

Seidel et al. [41] investigated empathic competencies in 30 incarcerate violent offenders and 30 healthy controls, using an experimental approach. They divided empathy into three core components; emotion recognition, perspective taking and affective responsiveness, and assessed these using three tasks [41]. Group difference findings, indicated a significant difference in accuracy at recognising facial emotional expressions between violent offenders and a control group, with violent offenders demonstrating impairments particularly with disgust stimuli. No significant differences were identified between groups in their accuracy at perspective taking or affective responsiveness. However, despite this, violent offenders with a higher number of past assaults, indicating more impulsive and recklessness traits, highlighted a trend of demonstrating lower accuracy in the perspective taking task. Limitations, identified by the authors, stressed low ecological validity, indicated via ceiling effects, in the affective responsiveness task and the importance for more naturalistic scenarios. Replication would also benefit from minimising the difficulty of the perspective taking task, as this evidenced floor effects across both groups, and extending the sample to include female offenders in order to investigate the link between empathy, gender and violence.

### Shorey et al. [42]

Shorey et al. [42] examined the association between impulsivity, trait anger and intimate partner aggression, psychological and physical, in a sample of 80 females mandated to attend a domestic violence intervention program. In addition to this, the authors researched whether these traits also linked to general aggression. Participants completed psychometrics that assessed for difficulties with impulse control, anger and acts of intimate partner violence. Results demonstrated that impulsivity predicted both physical aggression and trait anger, and that trait anger fully mediates the relationship between impulsivity and physical aggression by reducing the association of impulsivity and physical aggression. The exact same results were found for the mediation model of general aggression perpetration. However, methodological limitations, which would need to be addressed to increase the validity of future results include; the use of a cross-sectional rather than longitudinal design which prevented temporal associations, the reliance on self-report measures for impulsivity, rather than behavioural or performance-based measures which may capture more facets of this construct, and the use of a convenience sample which impairs generalisability of the findings to wider populations, such as a females in the community.

### Scott et al. [40]

Scott et al. [40] investigated whether emotion dysregulation may mediate diagnostic traits of BPD on psychological and physical aggression and victimization, after controlling for ASPD traits and impulsivity. The sample population consisted of 75 patients receiving treatment from a mental health outpatient’s clinic and 75 individuals in the community whom were not receiving mental health treatment. Participants consented to a clinical interview at baseline, completed a battery of self-report questionnaires at baseline and then at 3-monthly intervals over the year. Analysis suggested that emotion dysregulation but not impulsivity, significantly predicted physical assault perpetration over the course of the year. However BPD traits, including impulsivity itself, did not directly predict any form of aggression once the factor of emotion dysregulation was controlled for. ASPD traits were found to directly predict physical assault perpetration but not psychological aggression perpetration. The study had a number of limitations; the sample was unrepresentative of a treatment-seeking mental health population; only 9/150 met the diagnostic criteria for ASPD, of which, 4 of these individuals had co-morbid BPD diagnoses, as a result, the findings cannot be generalised to a population with severe personality traits nor criminal populations with a greater range of ASPD symptoms. Despite this, the wider findings suggest that emotion dysregulation may be a more effective predictor of physical and psychological aggression and victimization than trait impulsivity and diagnostic labels.

### Mauricio et al. [34]

Mauricio et al. [34] investigated whether a diagnosis of PD mediated the effects of insecure adult attachment on physical and psychological intimate partner violence in a population of 192 heterosexual males who had been court mandated to attend a community batterer intervention program. Participants completed self-report measures that assessed antisocial and borderline traits, attachment orientation, levels of physical violence and psychological violence towards intimate female partners, as well as a social desirability psychometric. Results identified that both ASPD and BPD diagnoses mediated the path between avoidant attachment and intimate partner violence and anxious attachment and intimate partner violence. Based on the findings, the authors suggest that work on interpersonal difficulties should be central in intimate partner violence therapeutic programs. A number of methodological limitations were noted, including cross-sectional design, monomeasure and monomethod bias. The study is further limited in its ability to draw in-depth conclusions, as it fails to consider the contribution of individual diagnostic traits to violence perpetration, instead relating violence to diagnoses as single constructs. Mauricio et al. [34] acknowledge that ASPDs and BPDs have commonalities due to being in the cluster B category, yet fail to detail the overlapping traits which may infer how both PD diagnoses mediate the relationship between both attachment orientations and physical violence.

### Fossati et al. [19]

Fossati et al. [19] utilised a sample of 461 outpatients from a psychological and psychotherapeutic PD service in Milan, to investigate whether or not personality and attachment traits were good distinguishers between the four Cluster B PDs identified within the Diagnostic and Statistical Manual of Mental Disorders-Fourth edition (DSM-IV, [2]). Analyses indicated that the trait of aggressiveness was related to both ASPDs and NPDs, but only the physical aspect of aggression was linked to ASPD. In comparison, NPD was only linked to the emotional traits of aggression, e.g. anger and irritability. In regards to BPD diagnosis, physical aggressive acts were deemed a result of poor impulse control, rather than a trait directly linked to the disorder itself, but impulsiveness itself was a distinguishing trait of BPD. This research highlights promising findings in regards to specific personality traits and physical aggression, but is flawed methodologically via sampling failures to include a control/comparison group, and utilising a specific outpatient population, which fails to represent those with more complex PD diagnoses. Additionally, findings were based on exploratory factor analysis only and as such, the authors themselves emphasised the need for a future cross-validation study using confirmatory factor analysis.

### Day et al. [9]

Day et al. [9] investigated the role of empathy in anger arousal within a sample of 51 violent offenders from a medium secure prison and a control group of 45 undergraduate students. Using an experimental method, participants watched a videotaped vignette of interpersonal events aimed at provoking anger and completed a self-predicted measure of anger in response to the scenario. Despite the assumption that violent offenders have deficits in their ability to take perspective and lack empathic concern, no significant differences were identified between violent offenders and students on these construct measures. However findings did indicate that for both groups, perspective taking was the strongest predictor of anger arousal in response to interpersonal provocation, as poor perspective taking skills exacerbated anger arousal. The study was limited by its small sample size, the reliance on self-report measures and thus the influence of social desirability, though acknowledged in the study as a confounding variable. Recommendations for future research included scrutiny of the influence of additional factors such as cognitive distortions on empathy processes and responses.

### Cunha & Gonçalves [7]

Cunha and Gonçalves [7] sought to identify distinct groups of intimate partner offenders by categorising according to psychopathology, violence severity and frequency. 187 batterers; 111 serving prison sentences and 76 serving community sentences, completed a battery of psychometric tests and a semi-structured clinical interview in order to aid hierarchical cluster analysis using Ward’s method [47]. The results of the cluster analysis supported Cunha and Goncalve’s prediction of distinct groups of batterer’s; non-pathological (40 % of this sample), antisocial/violent (27 % of this sample) and disturbed (33 % of this sample), and the concept of heterogeneity among males who commit intimate partner violence. Specifically, antisocial/violent group, as expected, exhibited higher scores on antisocial and psychopathic traits, such as lack of empathy and manipulation, and were significantly more violent in comparison to the other sub-groups. Disturbed batterer’s were significantly more physically ‘aggressive’ and appeared to experience higher levels of psychological distress, in particular on two psychopathology dimensions; depression and paranoid ideation. Cunha and Goncalves linked this sub-group of batterer’s to the “impulsive type” or “borderline type” as reported in previous literature. The study was limited by failure to include a comparison group (e.g. a non-violent population) and the use of typological analysis, which uses only a limited set of measures to classify individuals into groups.

### James & Seager [27]

James and Seager [27] investigated whether impulsivity and schemas of a hostile world were good predictors of persistent violence, as measured by assault, in 40 male Canadian prisoners. Using a dichotic shadowing task and social vignettes to assess hypervigilance for hostile words and attributions, it was identified that hostility significantly correlated with a history of persistent violence. Interestingly, impulsivity did not correlate with the two schema measures, but did with a history of persistent violence. The criticisms of the study relate to the small sample size, which may have underpowered the main findings and the poor correlation between the two measures of hostile schemas, suggesting weak construct validity.

### Warren et al. [48]

Warren et al. [48] sought to further investigate the relationship between Axis II disorders and community and institutional violence among a prison cohort of 261 female inmates. Warren et al. [48] identified from a population of 802 inmates, 200 nonpsychotic women who met the criteria for one of the four Cluster B PDs and 50 nonpsychotic women who did not meet the criteria for a Cluster B PD. In addition to this, prison files and a psychometric were used to identify violent behaviours. A diagnosis of any Cluster B PD significantly predicted self-reported institutional violence, and the presence of NPD diagnosis significantly predicted current prison time for any violent crime, including and excluding homicide. Both BPD and ASPD diagnoses were predictive of self-reported institutional violence. Warren and South [49], cited previously in this review, later proceeded to investigate the functional link between Cluster B personality symptoms and violence.

## Discussion

The review highlights the complexity of the relationship between violence and a PD diagnosis, as well as the limitations of the current literature with regards to the functional link. Current research does infer a relationship, albeit it weak and gender biased. Research also shows that the aetiology of violent behaviour attributed to PD is low in comparison to other well-known risk factors such as substance abuse and lifetime severe mental illness [45] Table 2. Summarises the key findings related to the specific PD diagnostic traits.

**Table 2:** Summary of evidence

	Link with violence
Antisocial	
Social norms	No specific reference within literature.
Deceitfulness	No specific reference within literature.
Impulsivity	Related to more violent assaults [41]. Did not directly predict aggression once emotional dysregulation was controlled for [40]. Impulsivity related to history of persistent violence [27]
Aggressiveness	Disturbed batterers more physically aggressive [7]. Aggressiveness related to ASPD and NPD diagnoses. In BPD aggressiveness linked to poor impulse control. [19].
Reckless	Related to more violent assaults [41].
Irresponsibility	No specific reference within literature.
Lack remorse	No specific reference within literature.
Borderline	
Avoid abandonment	Avoidant attachment predicted severity of physical partner violence mediated by interpersonal difficulties [31]. Female offenders have greater number of traumatic experiences and less secure attachment styles [22]. ASPD and BPD diagnoses mediated path between by avoidant attachment intimate partner violence [34].
Unstable relationships	See ‘Avoid Abandonment’.
Identity disturbance	No specific reference within literature.
Impulsivity	See ‘Impulsivity’ under Antisocial diagnosis.
Suicidal behavior	No specific reference within literature.
Affective instability	Emotional Dysregulation predicted physical assault [40].
Emptiness	No specific reference within literature.
Anger	Impulsivity predicted physical aggression and trait anger. Trait anger mediates relationship between impulsivity and physical aggression. [42].
Paranoid ideation	Disturbed batterers higher levels of paranoid ideation [7].
Narcissistic	
Grandiose	Link between experience grandiose delusions and serious violence. Stronger link to violence when associated with elation or anger [49].
Fantasies of success	No specific reference within literature.
Is “special”	See ‘Grandiose’.
Excessive admiration	No specific reference within literature.
Sense of entitlement	Evidence of inflated sense of entitlement linked to violent offenders. When violated more likely to be violent [18]
Exploitative	No specific reference within literature.
Lacks empathy	Violent offenders have reduced ability to recognise facial emotions [41]. Poor perspective taking exacerbated anger arousal [9]. Violent offender have poorer empathy [7].
Envious	No specific reference within literature.
Arrogant	No specific reference within literature.

Most of the studies reviewed, failed to empirically research the individual Cluster B personality traits, and instead chose to consider diagnosis as a sole entity. As there can be many combinations of traits in order to meet the clinical cut-off for a diagnosis of each of the Cluster B PDs, there is a need to look in-depth at those traits that distinguish individuals with a who engage in violent behaviours from those who do not.

This literature search highlighted the lack of empirical studies investigating the link between pathological NPD traits and violent behaviour, despite it being anecdotally linked to violence risk management for some time. Warren and South [49] highlighted the significance of considering narcissistic traits in violence risk assessment, when there is a tendency for professionals to link violence solely with ASPD.

Key findings from the literature in relation to NPD traits indicate that delusions of a grandiose nature, when associated with elation or anger can present a direct pathway to serious violence [44]. This lends consideration to precipitating factors that can influence elation or anger, such as substance misuse for example, which is known to be associated with PD, violence, and aggression [37]. Emotion dysregulation could also precipitate elation or anger, which may account for violence perpetration in the context of an inflated sense of entitlement being or feeling violated, as identified by Fisher and Hall [18]. From a Schema-focussed perspective, a violated sense of entitlement could result in behavioural externalisation of aggression as a means to overcompensate for such feelings of entitlement [28]. Further insight could be taken from the tenuous relationship identified between impaired accuracy in perspective taking and traits impulsivity and recklessness [41]. Impaired perspective taking was also identified to exacerbate anger arousal [9], which may thus enhance risk of impulsive and/or reckless violence.

Trait “aggressiveness” was identified to relate to NPD, however this was distinguished to refer solely to the emotional trait of aggressiveness, being anger and irritability, as opposed to physical acts of aggression [19]. Despite this, NPD comorbid with other PDs, significantly enhances the risk of serious physical violence, particularly murder [49], which supports the inference that trait impulsivity, associated with ASPD and BPD, may present a significant elevating risk factor towards the perpetration of physical violence or aggression in the context of NPD traits. The trait of impulsivity has theoretical linkage to personality structure as well as aggressive or violent behaviour [29]. In fact, Elonheimo et al. [15] discussed how they felt “violence may be attributed more to impulsiveness than actual mental disorder; it may arise out of situational factors, provocation, and an emotional surge”.

Research into violence associated with BPD was found to centre on domestic violence perpetration. In formulating violence associated with BPD traits, Goldenson et al. [22], identified that personal histories of trauma, commonly associated with dimensions of PDs [23], relate to less secure attachment styles, indicative of trait “avoid abandonment”. Of which, both avoidant and anxious attachment in the context of BPD or ASPD diagnoses relate to intimate partner violence [34] and avoidant attachment was predictive of the severity of intimate partner violence [31]. Emotion dysregulation was identified to be a superior predictor of assault perpetration, physical and psychological aggression, than trait impulsivity [40], indicating that trait “affective instability” is an important contributory factor to interpersonal difficulties and resultant violence or aggression. The influence of affective instability on violence is further supported by Shorey et al. [42], as despite impulsivity being a predictor of aggression and trait anger, the finding that affective trait anger mediates the relationship between impulsivity and physical aggression, along with the finding that higher levels of psychological distress, psychopathologised as depression and paranoid ideation, related to more physically aggressive behaviour [7] suggest the affective accountability and relevance to violence perpetration. Nevertheless, trait impulsivity, though likely precipitated by affective instability, remains to present a risk factor as it has been deemed to account for acts of physical aggression in the context of BPD by Fossati et al. [19].

ASPD symptoms were reported to directly predict greater physical assault perpetration and victimization and were not associated with difficulties regulating emotions [40]. Hostile attributions and trait impulsivity have been shown to correlate with a history of persistent violence in the context of ASPD [27] and Crick and Dodge [6] have proposed a model that emphasizes that violence occurs as a result of a chain of events propelled initially by making hostile attributions.

The literature identifies a clear overlap between traits and/or symptoms across PD diagnoses and offers some insight into the relevance of trait presence, comorbidity and interaction in the formulation and prediction risk of violence. It is therefore important to be mindful of a number of difficulties when it comes to the relationship between risk of violence and offenders with PDs, in order not to attribute unrealistic weighting to PDs in risk management. Firstly, it is likely that violence is overestimated as a risk in PDs as a whole, due to confounding variables such as sociodemographic factors and co-morbidity with other Axis II disorders, Axis I disorders and substance abuse [1]. In fact, Tyrer et al. [43] refers to the “morass of comorbidity” as holding the key to the causal relationship between PD and violence.

It is perhaps in scrutinising this concept of comorbidity, that insight can be elicited in regard to the complex interactions between specified traits that may point towards a more idiographic approach to risk management. Though the research is not presently at a point to be comprehensive, certain patterns are emerging in relation to the presence and dynamic interaction of diagnostic traits which point towards a preliminary model that may be more relevant to clinicians in assessing and managing violence with individual PD diagnoses. The presence of such diagnostic traits for individuals does not always nor consistently result in violent behaviour, which further complicates their accountability for risk attribution in assessment. It is proposed then, that in formulating risk, the presence of such traits may serve as predisposing factors which, when precipitated by their idiosyncratic interaction, emotional arousal or dysregulation, interpersonal difficulties and or impulsivity, which were common features in the literature, risk of presenting violence may be predicted.

**Fig. 2:**
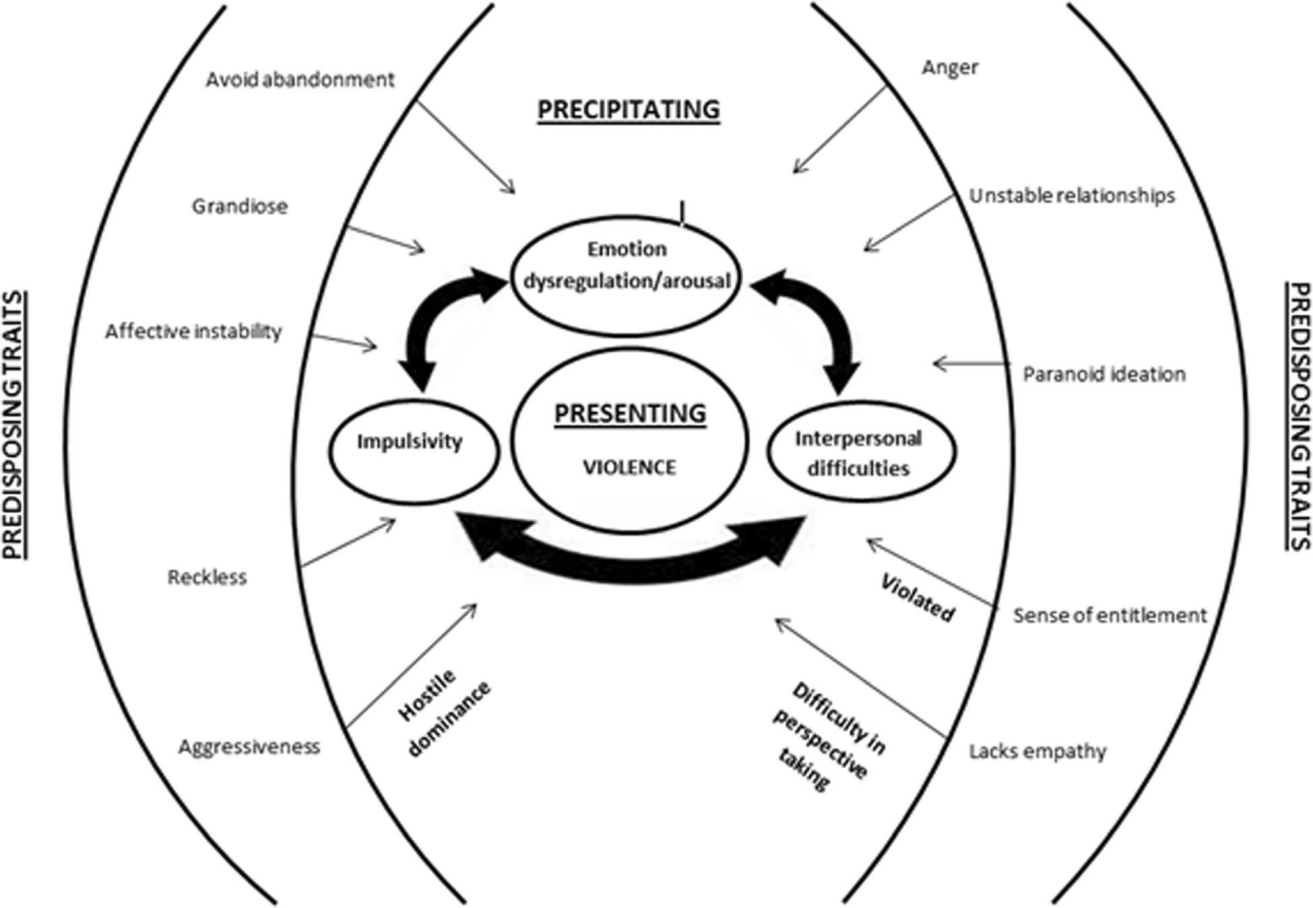
Violence formulation model

As a preliminary attempt to illustrate this, Fig. 2, places violence, as previously defined via the HCR-20, at the centre of the model as the presenting problem. The next layer depicts the three main contributory factors that the literature indicates may precipitate violence (emotion dysregulation/arousal, interpersonal difficulties and impulsivity); all of which are proposed to influence one another non-directionally with an aggregate effect. The degree to which these factors interact (and thus result in violence) however, depends on the precipitating traits that are present, or that have been triggered, and their interaction with one another. It is therefore the interplay of the predisposing traits which influence the degree to which precipitating factors aggregate to present in violent behaviour.

In explaining the inconsistency in presenting violent behaviour, the presence of predisposing PD traits may exist and interact with precipitating factors in a dysfunctional yet non-violent manner, but may escalate in risk on interacting or being augmented by further traits. For example, an individual with the predisposing trait “unstable relationships” may experience chronic interpersonal difficulties that may have the potential to precipitate violence, without violence occurring. It may only be when the predisposing trait “avoid abandonment” is triggered in addition to “unstable relationships” that interpersonal difficulties, emotional dysregulation and/or impulsivity may escalate and aggregate to result in violence perpetration. This may account for domestic violence that was deemed to be of the “impulsive type” or “borderline” type described by Cunha and Gonçalves [7]. An alternative example to illustrate this could be of an individual with the predisposing trait “sense of entitlement”. It is only when this is triggered by a sense of feeling violated that violence may occur as concluded by Fisher and Hall [18].

This model emphasises that individuals may present with any of the predisposing traits and not exhibit violence. It is instead the interplay of the predisposing traits with the precipitating factors that amount to an increase in risk of violence. As such, formulations of violence in the context of PD diagnoses should be very context specific, considering how each trait may or may not independently and collectively result in violence. This supports and highlights the importance of idiosyncratic formulation in structured professional judgement risk assessments, as used in the HCR-20; which considers previous violence, the specific individual factors present and their interplay, as opposed to a standardised checklist approach, more fitting with an actuarial risk approach.

Idiosyncratic risk factors are vulnerable to changes in circumstance which can subsequently impact on an increase and decrease in risk. With the first example mentioned earlier, the loss of a loved one or a breakdown in relationship may increase risk of violence via triggering “avoid abandonment” and subsequent emotional arousal, whereas stable relationships may be protective against such vulnerability and minimise the risk of violence. Increased support in the areas of idiosyncratic risk factors can therefore impact on managing levels of risk and enhancing dynamic risk management under changes in circumstance. Transparent, collaborative assessment and formulation of idiosyncratic risk factors can raise service user insight and awareness, enhance trust and working alliance [25], support their ongoing self-management and consequentially minimise risk of violence towards others.

### Recommendations

The evidence base surrounding Cluster B PDs and risk is presently limited. Evidence is also sparse regarding specific Cluster B NPD traits, which could be further researched. This review proposes a theoretical model for formulating risk of violence, which may be enhanced via future replication of the present methodology in reviewing Cluster B diagnostic traits and risk in the context of sexual violence. The review highlights the importance of idiosyncratic risk factors associated with PD diagnostic traits, which could provide a theoretical basis for further development of PD specific violence risk assessment.

It should be noted that a large majority of the studies included in this review involve individuals who have already been involved in violence. This makes it difficult to discount the impact of financial, social and cultural factors that may impact on the likelihood of violence and reflects the relative dearth of literature currently available linking specific PD symptoms and violence. Further research within different social/subcultural settings not related to violence would be recommended.

## Conclusions

The present review highlights considerable inconsistencies in assessing the influence of Cluster B PD diagnoses on risk of violence. Comorbidity in diagnoses presents an additional complexity in risk assessment, as evidence suggests that comorbidity is deemed to enhance risk of violence, yet fails to determine a theoretical basis for this.

In the future, in order to assess the level of risk of violence posed by an offender with personality difficulties, it is imperative to look at personality traits or maladaptive behaviours, in line with changes in DSM-IV, rather than the categorical nature of the disorder. This is due to the fact that personality traits have proven to be stronger predictors of violence than PDs. For example, increased ‘symptoms’ of DSM-IV Cluster A or Cluster B disorders correlate significantly with violence [17]. The review presents a model for formulating idiosyncratic risk of violence via consideration of the presence of diagnostic traits and the risk vulnerabilities posed by their interaction.

## Abbreviations

ASPD: Antisocial personality disorder
BPD: Borderline personality disorder
DSM-IV: Diagnostic and statistical manual of mental disorders-fourth edition
NPD: Narcissistic personality disorder
PD: Personality disorder
SOEQ: Sense of entitlement questionnaire’
